# Supplementary material for: Oxytocin via oxytocin receptor excites neurons in the endopiriform nucleus of juvenile mice
Source: Sci Rep. 2022 Jul 6;12:11401. doi: 10.1038/s41598-022-15390-5 (PMC9259672; doi:10.1038/s41598-022-15390-5)
Supplement: Supplementary file 1 — Supplementary Information. [file 41598_2022_15390_MOESM1_ESM.pdf]

Supplementary material for "Oxytocin via oxytocin receptor excites neurons in the endopiriform nucleus of juvenile mice"

Authors: Lindsey M. Biggs and Elizabeth A.D. Hammock

Supplementary Table 1: Statistical analyses. Table shows the statistical analyses used for each figure in the main text. Data was analyzed at the level of the individual cell. In some cases, data from more than one cell was collected per animal so the number and sex of animals included in the sample size is included in the table. Once an experimental bath was applied to the slice, it was discarded so all cells were naïve to the experimental drug baths at the start of the recording.

	Figure #	Data Structure	Statistical test	Sample Size	Statistical Results	Post-hoc test results
A	4A	Normal Distribution	One-way RM ANOVA with Geisser-Greenhous correction (GG)	n = 6 cells, 3 animals: 1 male, 2 female	F = 6.315, p = 0.015	Dunnett MCT: Baseline vs OXT 4 min: p = 0.011, d = 1.87; Baseline vs OXT 6 min: p = 0.253; Baseline vs Washout: p = 0.768
B	4B	Normal Distribution	One-way RM ANOVA with GG correction	n = 8 cells, 2 animals: 1 male, 1 female	F = 8.918, p = 0.008	Dunnett MCT: Baseline vs OXT 4 min: p = 0.036, d = 1.25; Baseline vs OXT 6 min: p = 0.246; Baseline vs Washout: p = 0.014, d = 1.35
C	4D	Normal Distribution	One-way RM ANOVA with GG correction	n = 11 cells, 4 animals: 2 male, 2 female	F = 6.165, p = 0.006	Dunnett MCT: Baseline vs OXT 4 min: p = 0.019, d = 0.275; Baseline vs OXT 6 min: p = 0.022, d = 0.379; Baseline vs Washout: p = 0.72
D	4E	Normal Distribution	One-way RM ANOVA with GG correction	n = 11 cells, 4 animals: 2 male, 2 female	F = 5.533, p = 0.014	Dunnett MCT: Baseline vs OXT 4 min: p = 0.395; Baseline vs OXT 6 min: p = 0.001, d = 0.518; Baseline vs Washout: p = 0.682
E	4F	Normal Distribution	One-way RM ANOVA with GG correction	n = 11 cells, 4 animals: 2 male, 2 female	F = 5.426, p = 0.018	Dunnett MCT: Baseline vs OXT 4 min: p = 0.003, d = 1.185; Baseline vs OXT 6 min: p = .009, d = 1.739; Baseline vs Washout: p > 0.999
F	4K	Normal Distribution	One-way RM ANOVA with GG correction	n = 10 cells, 6 animals: 4 male, 2 female	F = 2.628, p = 0.107	NA
G	4L	Normal Distribution	One-way RM ANOVA	n = 10 cells, 6 animals:	F = 0.194, p = 0.753	NA

			with GG correction	4 male, 2 female		
H	4M	Normal Distribution	One-way RM ANOVA with GG correction	n = 10 cells, 6 animals: 4 male, 2 female	F = 0.778, p = 0.447	NA
I	5A	Normal Distribution	One-way RM ANOVA with GG correction	n = 13 cells, 5 animals: 3 male, 2 female	F = 6.388, p = 0.005	Dunnett MCT: Baseline vs OXT 4 min: p = 0.0497, d = 0.444; Baseline vs OXT 6 min: p = 0.021, d = 0.606, Baseline vs Washout: p = 0.999
J	5B	Normal Distribution	One-way RM ANOVA with GG correction	n = 13 cells, 5 animals: 3 male, 2 female	F = 5.443, p = 0.019	Dunnett MCT: Baseline vs OXT 4 min: p = 0.043, d = 0.243; Baseline vs OXT 6 min: p = 0.007, d = 0.347; Baseline vs Washout: p = 0.997
K	5C	Normal Distribution	One-way RM ANOVA with GG correction	n = 13 cells, 5 animals: 3 male, 2 female	F = 4.868, p = 0.025	Dunnett MCT: Baseline vs OXT 4 min: p = 0.001, d = 0.53; Baseline vs OXT 6 min: p = 0.001, d = 0.753; Baseline vs Washout: p = 0.928
L	6A	Normal Distribution	One-way RM ANOVA with GG correction	n = 19 cells, 8 animals: 3 male, 5 female	F = 19.5, p < 0.0001	Dunnett MCT: Baseline vs OXT 4 min: p = 0.004, d = 0.404; Baseline vs OXT 6 min: p = 0.0002, d = 0.407; Baseline vs Washout: p = 0.02, d = 0.409
M	6B	Normal Distribution	One-way RM ANOVA with GG correction	n = 19 cells, 8 animals: 3 male, 5 female	F = 6.342, p = 0.01	Dunnett MCT: Baseline vs OXT 4 min: p = 0.017, d = 0.166; Baseline vs OXT 6 min: p = 0.017, d = 0.202; Baseline vs Washout: p = 0.447

Supplementary Table 2: Oxytocin effect on the number of spontaneous action potentials (APs) in cells held near threshold. Data presented as the absolute change from baseline value. For each cell, the baseline number of spontaneous action potentials was subtracted from the number of spontaneous action potentials during the experimental bath indicated. Data used for analysis of Figure 4A.

Cell ID	Baseline (normalized to 0)	4 min: Δ# of AP (4min-baseline)	6 min: Δ# of AP (6min-baseline)	Washout (30 min): Δ# of AP (Washout-baseline)
03182019-2-1	0	30	49	-9
03212019-1-1	0	57	69	5
03252019-2-1	0	42	-19	-28
03182019-2-3	0	27	36	24
03212019-1-2	0	7	-3	-3
03252019-2-2	0	34	19	-29

Supplementary Table 3: TGOT effect on the number of spontaneous APs in cells held near threshold. Data presented as the absolute change from baseline value. For each cell, the baseline number of spontaneous APs was subtracted from the number of spontaneous APs during the experimental bath indicated. Data used for analysis of Figure 4B.

Cell ID	Baseline (normalized to 0)	4 min: Δ# of AP (4min-baseline)	6 min: Δ# of AP (6min-baseline)	Washout: Δ# of AP (Washout-baseline)
04242019-1-1	0	-3	-3.5	-4.5
04242019-1-2	0	21	13	-1.5
04242019-1-3	0	37	23.5	-11.5
10042019-2-1	0	15.5	20	-6.5
04242019-1-5	0	29	6.5	-3.5
04242019-1-6	0	43.5	64.5	-7
10042019-2-2	0	24.5	8.5	-18
10042019-2-3	0	-6.5	-13	-17.5

Supplementary Table 4: TGOT effect on the resting membrane potential (RMP) of cells with no holding current applied. Data presented as the absolute change from baseline value. For each cell, the baseline RMP was subtracted from the RMP during the experimental bath indicated. Data used for analysis of Figure 4D.

**Cell ID**	**Baseline RMP (normalized to 0)**	**4 min: ΔRMP (mV) (4min-Baseline)**	**6 min: ΔRMP (mV) (6min-Baseline)**	**Washout: ΔRMP (Washout -Baseline)**
05282019-1-3	0	0.696014404	2.616638184	1.485603333
05292019-2-3	0	2.827163696	0.539176941	1.483146667
05292019-2-4	0	1.646781921	6.568389893	0.46572113
05312019-1-1	0	1.642494202	2.380065918	5.337211609
05282019-1-1	0	2.591697693	1.735923767	-3.197929382
05282019-1-4	0	1.636474609	3.605308533	1.578430176
05292019-2-1	0	4.82686615	10.16745758	1.4323349
05292019-2-2	0	-0.624221802	1.161521912	-2.684371948
05302019-1-3	0	0.140625	0.165588379	-2.010498047
05312019-1-3	0	7.757534027	7.926109314	1.602947235
05312019-1-4	0	2.710834503	0.039001465	1.597640991

Supplementary Table 5: TGOT effect on the membrane resistance (MΩ) of cells with no holding current applied. Data presented as the absolute change from baseline value. For each cell, the baseline membrane resistance was subtracted from the membrane resistance during the experimental bath indicated. Data used for analysis of Figure 4E.

**Cell ID**	**Baseline Resistance (MΩ) (normalized to 0)**	**4 min: ΔResistance (MΩ) (4min-Baseline)**	**6 min: ΔResistance (MΩ) (6min-Baseline)**	**Washout: ΔResistance (MΩ) (Washout-Baseline)**
05282019-1-3	0	14.58374023	52.74810791	82.5579834
05292019-2-3	0	10.28945923	67.52059937	7.488098145
05292019-2-4	0	36.64117432	160.4873047	-27.64929199
05312019-1-1	0	35.64562988	59.28649902	89.65576172
05282019-1-1	0	-29.7315979	43.52920532	87.12939453
05282019-1-4	0	17.10968018	14.79064941	-51.74697876
05292019-2-1	0	24.2539978	96.58425903	18.02429199
05292019-2-2	0	15.22015381	68.0960083	-2.096740723
05302019-1-3	0	10.78018188	20.47210693	-9.259796143
05312019-1-3	0	125.8762512	92.5930481	-33.42858887
05312019-1-4	0	-52.49676514	38.06970215	-4.746551514

Supplementary Table 6: TGOT effect on the number of APs evoked by a two second intracellular current injection. Value of current injection was determined for each cell prior to the start of data collection such that multiple APs were evoked (between 2-10 evoked APs) to avoid floor and ceiling effects. Data presented as the absolute change from baseline value. For each cell, the baseline number of evoked APs was subtracted from the number of APs during the experimental bath indicated. Data used for analysis of Figure 4F.

Cell ID	Baseline # of evoked AP (normalized to 0)	4 min: Δ # of evoked AP (4min-Baseline)	6 min: Δ # of evoked AP (6min-Baseline)	Washout: Δ # of evoked AP [Washout -Baseline]
05282019-1-3	0	0	1	3
05292019-2-3	0	5	6	-2
05292019-2-4	0	1	-1	1
05312019-1-1	0	3	5	6
05282019-1-1	0	3	0	-1
05282019-1-4	0	1	6	1
05292019-2-1	0	6	5	-3
05292019-2-2	0	2	6	7
05302019-1-3	0	7	12	4
05312019-1-3	0	6	6	-6
05312019-1-4	0	2	1	-10

Supplementary Table 7: TGOT effect on the RMP (mV) of cells from OXTR-KO tissue with no holding current applied. Data presented as the absolute change from baseline value. For each cell, the baseline RMP was subtracted from the RMP during the experimental bath indicated. Data used for analysis of Figure 4K.

Cell ID	Baseline RMP (mV) (normalized to 0)	4 min: ΔRMP (mV) (4min-Baseline)	6 min: ΔRMP (mV) (6min-Baseline)
12232019-1-1	0	-0.693328857	-1.93396759
12232019-1-3	0	-2.166801453	-0.376533508
12232019-1-4	0	0.391983032	-0.35471344
01072020-1-3	0	-7.566902161	-7.031013489
01082020-1-2	0	-3.481842041	1.260932922
01082020-1-3	0	-6.31060791	-4.068275452
01102020-1-1	0	0.793952942	-2.843727112
01102020-1-2	0	1.096287964	0.381650208
01132020-1-2	0	-0.200508118	0.748931885
02042020-1-1	0	-0.159294128	0.038200378

Supplementary Table 8: TGOT effect on the membrane resistance (MΩ) of OXTR-KO cells with no holding current applied. Data presented as the absolute change from baseline value. For each cell, the baseline membrane resistance was subtracted from the membrane resistance during the experimental bath indicated. Data used for analysis of Figure 4L.

Cell ID	Baseline Resistance (MΩ) (normalized to 0)	4 min: ΔResistance (MΩ) (4min-Baseline)	6 min: ΔResistance (MΩ) (6min-Baseline)
12232019-1-1	0	3.869628906	-10.13870239
12232019-1-3	0	-71.50524902	-12.82406616
12232019-1-4	0	14.22576904	0.046234131
01072020-1-3	0	-62.29217529	-74.67697144
01082020-1-2	0	13.78967285	22.7479248
01082020-1-3	0	26.79199219	-0.336303711
01102020-1-1	0	71.8230896	49.43664551
01102020-1-2	0	-76.90200806	-34.25872803
01132020-1-2	0	4.048309326	4.122924805
02042020-1-1	0	2.083282471	21.20849609

Supplementary Table 9: TGOT effect on the number of APs evoked by a two second intracellular current injection in OXTR-KO cells. Value of current injection was determined for each cell prior to the start of data collection such that multiple APs were evoked (between 2-12 evoked APs) to avoid floor and ceiling effects. Data presented as the absolute change from baseline value. For each cell, the baseline number of evoked APs was subtracted from the number of APs during the experimental bath indicated. Data used for analysis of Figure 4M.

Cell ID	Baseline # of evoked AP (normalized to 0)	4 min: Δ # of evoked AP (4min-Baseline)	6 min: Δ # of evoked AP (6min-Baseline)
12232019-1-1	0	1	2
12232019-1-3	0	1	0
12232019-1-4	0	1	2
01072020-1-3	0	-3	-2
01082020-1-2	0	0	0
01082020-1-3	0	-5	-4
01102020-1-1	0	-1	-3
01102020-1-2	0	0	0
01132020-1-2	0	0	2
02042020-1-1	0	-1	1

Supplementary Table 10: TGOT effect on the RMP of cells under glutamate and GABA receptor blockade (DNQX:5 µM, APV: 10 µM, picrotoxin:200 nM) with no holding current applied. Data presented as the absolute change from baseline value (mV). For each cell, the baseline RMP was subtracted from the RMP during the experimental bath indicated. Data used for analysis of Figure 5A.

**Cell ID**	**Baseline RMP (mV) (normalized to 0)**	**4 min: ΔRMP (mV) (4min-Baseline)**	**6 min: ΔRMP (mV) (6min-Baseline)**	**Washout: ΔRMP (mV) (Washout-Baseline)**
05132019-1-4	0	2.614860535	4.441215515	-3.023742676
05142019-1-2	0	1.037597656	2.560382843	-0.432037354
05092019-1-2	0	10.62329102	13.20077133	7.15184021
05102019-1-1	0	8.972099304	14.1973381	11.76184464
05102019-1-2	0	0.878650665	1.97864151	-8.608024597
05102019-1-3	0	12.60706711	11.95859528	-2.652645111
05152019-1-2	0	-0.470970154	2.177902222	-1.611412048
05152019-1-3	0	1.394363403	4.136558533	-2.614006042
05132019-1-2	0	0.414199829	0.590335846	-2.006576538
05142019-1-3	0	3.685409546	4.94493866	1.160240173
05142019-1-4	0	-1.000091553	-1.991653442	3.716644287
05102019-1-4	0	4.070259094	3.790687561	-3.143341064
05152019-1-4	0	-0.903282166	-1.537826538	1.455093384

Supplementary Table 11: TGOT effect on the membrane resistance (MΩ) of cells under glutamate and GABA receptor blockade (DNQX:5 µM, APV: 10 µM, picrotoxin:200 nM) with no holding current applied. Data presented as the absolute change from baseline value. For each cell, the baseline membrane resistance was subtracted from the membrane resistance during the experimental bath indicated. Data used for analysis of Figure 5B.

**Cell ID**	**Baseline Resistance (MΩ) (normalized to 0)**	**4 min: ΔResistance (MΩ) (4min-Baseline)**	**6 min: ΔResistance (MΩ) (6min-Baseline)**	**Washout: ΔResistance (MΩ) (Washout-Baseline)**
05132019-1-4	0	32.47894287	70.64300537	-124.1886749
05142019-1-2	0	20.34307861	19.85501099	14.40634155
05092019-1-2	0	154.1160889	216.7189941	73.88269043
05102019-1-1	0	11.41659546	56.56439209	43.40658569
05102019-1-2	0	62.35211182	90.87200928	-139.1866455
05102019-1-3	0	178.5906982	173.4503784	-9.690093994
05152019-1-2	0	-12.47467041	23.2482605	16.49533081
05152019-1-3	0	42.41680908	34.14300537	-5.99822998
05132019-1-2	0	2.28112793	4.505859375	16.2361145
05142019-1-3	0	37.98736572	46.48522949	32.37243652
05142019-1-4	0	-12.37243652	43.93432617	112.7546387
05102019-1-4	0	60.81539917	69.62713623	8.422698975
05152019-1-4	0	11.22344971	12.32666016	1.267486572

Supplementary Table 12: TGOT effect on the number of APs evoked by a two second intracellular current injection under glutamate and GABA receptor blockade (DNQX:5 µM, APV: 10 µM, picrotoxin:200 nM). Value of current injection was determined for each cell prior to the start of data collection such that multiple APs were evoked (between 2-10 evoked APs) to avoid floor and ceiling effects. Data presented as the absolute change from baseline value. For each cell, the baseline number of evoked APs was subtracted from the number of APs during the experimental bath indicated. Data used for analysis of Figure 5C.

Cell ID	Baseline # of evoked AP (normalized to 0)	4 min: Δ # of evoked AP (4min-Baseline)	6 min: Δ # of evoked AP (6min-Baseline)	Washout: Δ # of evoked AP [Washout-Baseline]
05132019-1-4	0	2	9	-1
05142019-1-2	0	4	3	-8
05092019-1-2	0	5	4	-2
05102019-1-1	0	1	6	2
05102019-1-2	0	3	5	-6
05102019-1-3	0	3	1	-1
05152019-1-2	0	4	16	11
05152019-1-3	0	1	3	-5
05132019-1-2	0	3	10	0
05142019-1-3	0	10	10	2
05142019-1-4	0	5	8	25
05102019-1-4	0	9	5	0
05152019-1-4	0	0	0	-2

Supplementary Table 13: TGOT effect on the RMP (mV) of cells in the presence of TTX (1 µM) with no holding current applied. Data presented as the absolute change from baseline value. For each cell, the baseline RMP was subtracted from the RMP during the experimental bath indicated. Data used for analysis of Figure 6A.

Cell ID	Baseline RMP (mV) (normalized to 0)	4 min: ΔRMP (mV) (4min-Baseline)	6 min: ΔRMP (mV) (6min-Baseline)	Washout: ΔRMP (mV) [Washout-Baseline]
06192019-1-2	0	2.076293945	1.349533081	-3.132415771
06192019-1-3	0	7.475727081	7.475727081	-0.714347839
06202019-1-2	0	2.156536102	1.41431427	-3.007465363
06212019-1-3	0	1.830680847	3.22858429	-0.199333191
06212019-1-4	0	0.898830414	2.613349915	3.324886322
06242019-1-5	0	0.910942078	0.641773224	-2.198093414
06282019-1-3	0	1.722602844	1.731731415	-4.561096191
07032019-1-2	0	5.011901855	1.627258301	-0.368354797
07022019-1-3	0	3.097732544	3.07056427	-2.159843445
07092019-1-1	0	3.499336243	2.659255981	2.383552551
07092019-1-3	0	-0.939495087	0.839252472	1.221614838
06282019-1-2	0	0.496017456	3.161933899	-4.170066833
07032019-1-3	0	-0.770851135	-0.426704407	-4.017044067
07022019-1-2	0	5.854957581	2.937576294	-5.558082581
06202019-1-4	0	2.425674438	0.375320435	2.910491943
06192019-1-1	0	0.205818176	2.624984741	-8.275993347
06202019-1-3	0	-0.740913391	1.056350708	-3.241455078
06212019-1-2	0	0.606632233	1.475036621	-4.141872406
06242019-1-4	0	1.357658386	0.27570343	-8.621170044

Supplementary Table 14: TGOT effect on the membrane resistance (MΩ) of in the presence of TTX (1 µM) with no holding current applied. Data presented as the absolute change from baseline value. For each cell, the baseline membrane resistance was subtracted from the membrane resistance during the experimental bath indicated. Data used for analysis of Figure 6B.

Cell ID	Baseline Resistance (MΩ) (normalized to 0)	4 min: ΔResistance (MΩ) (4min-Baseline)	6 min: ΔResistance (MΩ) 6min-Baseline)	Washout: ΔResistance (MΩ) (Washout-Baseline)
06192019-1-2	0	-6.655578613	1.72076416	-26.00891113
06192019-1-3	0	107.2595215	107.2595215	-122.4525146
06202019-1-2	0	98.52502441	148.8198242	86.13128662
06212019-1-3	0	37.29089355	55.46832275	-29.31195068
06212019-1-4	0	10.63800049	47.73400879	68.54412842
06242019-1-5	0	72.60980225	58.23278809	-36.38931274
06282019-1-3	0	35.83868408	16.54803467	-20.11120605
07032019-1-2	0	-38.24719238	28.96051025	123.1519775
07022019-1-3	0	45.42984009	74.36080933	-1.886749268
07092019-1-1	0	21.24310303	-9.571533203	-30.16619873
07092019-1-3	0	5.35484314	11.75788879	7.953643799
06282019-1-2	0	21.74603271	52.8343811	37.30969238
07032019-1-3	0	-4.851074219	6.173553467	-55.82244873
07022019-1-2	0	59.34356689	56.72393799	-95.28070068
06202019-1-4	0	28.89868164	32.00027466	37.44299316
06192019-1-1	0	-27.77496338	-53.84738867	-162.2638245
06202019-1-3	0	7.100219727	3.007324219	28.53436279
06212019-1-2	0	29.0201416	3.15826416	-128.7002563
06242019-1-4	0	8.290557861	-14.51782227	-118.9764099
